# Association Between Reduced Daily Steps and Sarcopenic Obesity in Treatment-Seeking Adults With Obesity

**DOI:** 10.3389/fendo.2020.00022

**Published:** 2020-01-30

**Authors:** Dima Kreidieh, Leila Itani, Dana El Masri, Hana Tannir, Marwan El Ghoch

**Affiliations:** Department of Nutrition and Dietetics, Faculty of Health Sciences, Beirut Arab University, Beirut, Lebanon

**Keywords:** physical activity, body composition, obesity, sarcopenic obesity, daily steps, sedentary lifestyle

## Abstract

**Objectives:** Understanding the condition that describes the coexistence of obesity and sarcopenia, termed sarcopenic obesity (SO), is becoming a scientific and clinical priority. In this study, we aimed to assess the prevalence of SO in treatment-seeking adults with obesity and investigate any potential association between SO and a sedentary lifestyle, expressed in terms of daily steps.

**Methods:** In this cross-sectional, prospective observational study, body composition and daily steps measurements were obtained using a segmental body composition analyser (Tanita BC-418) and an Omron HJ-320 pedometer, respectively, in 111 adults of both genders with obesity (body mass index; BMI ≥ 30 kg/m^2^), referred to the Outpatient Clinic in the Department of Nutrition and Dietetics at Beirut Arab University (BAU) in Lebanon. The participants were then categorized according to the presence of absence of SO, defined as an appendicular lean mass divided by body weight (ALM/weight) × 100%) of less than 23.40 and 29.60 in females and males, respectively.

**Results:** Fifty-five of the 111 participants with obesity, with a mean age of 39.62 ± 16.55 years and a mean BMI of 38.05 ± 5.33 kg/m^2^ met the criteria for SO and displayed a significantly higher prevalence of inactivity (<5,000 daily steps), i.e., nearly double (54.5% vs. 32.1%; *p* = 0.017) and they had a lower mean number of daily steps than those in the group without SO (5,279 ± 2,641 vs. 6,732 ± 2,989; *p* = 0.008). Linear regression analysis showed that SO is associated with a lower number of daily steps by 1,421 (β = −1421.4; −2508.9, −333.9; *p* = 0.011) after adjusting for age, gender employment and the presence of cardiometabolic disease.

**Conclusion:** Sarcopenic obesity affects nearly 50% of treatment-seeking adults with obesity. Moreover, it seems to be associated with a lower number of daily steps and a sedentary lifestyle. Future studies are needed to clarify whether this may influence clinical outcomes. If this is shown to be the case, weight management programmes should incorporate additional physical activity strategies in this population.

## Introduction

A new phenotype has been identified, which occurs in the presence of both sarcopenia and obesity, and is termed sarcopenic obesity (SO), describing the coexistence of increased body-fat mass deposition and a reduction in lean mass as well as muscle strength (1, 2). Understanding of this condition is now becoming a scientific and clinical priority (1). Indeed, several studies have demonstrated that regardless of gender, individuals with SO have worse profiles in terms of cardio-metabolic conditions (i.e., hyperglycaemia, hypertension, dyslipidaemia, insulin resistance, and type 2 diabetes) (3–6). However, the underlying mechanism is still unclear, but it seems there is a bi-directional interaction between “obesity” and “sarcopenia” through a further enhancement of chronic inflammation levels (i.e., common denominator seen in both conditions) (7, 8), that appears to exacerbate the cardiometabolic comorbidities (9) in individuals affected by SO, rather than obesity alone.

In the same direction, interestingly, recent studies have found a strong association between SO and impairment of physical performance (10). However, to the best of our knowledge, it is still unclear whether individuals with SO are likely to have a more sedentary lifestyle than those with obesity alone, however, if this is true, it may have significant clinical implications, especially among those seeking treatment and in the identification of SO in patients with obesity, for whom it becomes critically important to target interventions (11).

Definitions of SO based only on lean mass and physical fitness without accounting for body mass (i.e., body weight, BMI, etc.) may be strongly skewed for at least two reasons (6, 12–14). Firstly, patients with obesity tend to have a relatively large lean mass (15). Hence, sarcopenia criteria may not be met in these individuals, and the prevalence of sarcopenia may be underestimated (16). Second, low physical fitness is more closely associated with obesity than sarcopenia (17).

Based on these considerations, we aimed to assess the prevalence of sarcopenia in treatment-seeking adults of both genders with obesity, using a definition that, in addition to appendicular lean mass (ALM), also includes body weight (18), namely the definition proposed by Oh and colleagues from previous studies, which has been determined to be more clinically useful than other definitions (3, 19). Moreover, we also sought to examine any association between SO and a sedentary lifestyle—expressed as a reduction in the number of daily steps—when compared with those without SO.

## Materials and Methods

### Subjects

A priori calculation of the sample size showed that a sample of 92 patients is need for a power of 80% with medium effect size and 5 predictors. However, 111 participants of both genders seeking weight-loss treatment and with obesity were recruited consecutively, following referral by general practitioners to the Nutritional and Weight Management Outpatient Clinic in the Department of Nutrition and Dietetics at Beirut Arab University (BAU) in Lebanon during the period May 2017 to July 2019. The eligibility criteria were assessed by a medical doctor involved in the study and comprised: age ≥ 18 years, with a BMI ≥ 30.0 kg/m^2^, indicative of obesity status and considered a criterion for an absolute indication for weight loss (20). There were no specific exclusion criteria, except pregnancy or lactation, medication known to influence body weight or composition, or any clinical condition that does not indicate weight loss. The study was approved by the Institutional Review Board of BAU (No. 2017H-0034-HS-R-0241), and all participants gave informed, written consent for the use of their anonymized personal data. A questionnaire was administered to participants and controls in the test to elicit information regarding medical history, lifestyle, demographic and social conditions.

### Methods

Body weight was measured by trained dieticians using an electronic weighing scale (SECA 2730-ASTRA, Germany). Height was measured using a stadiometer. BMI was then calculated according to the standard formula.

Body composition was measured in the morning in our clinics by trained dieticians using a segmental body composition analyser (BC-418, Tanita Corp., Tokyo, Japan). Participants wore their own clothes and weight adjustment for clothing was applied. This method allows bioelectrical impedance measurement of the whole body and each part (right leg, left leg, right arm and left arm) at a single frequency. Gender, age and height information was then entered into the device and participants were asked to stand in a stable position with bare feet. Their toes and heels were placed in contact with the anterior and posterior electrodes of the weighing platform, respectively. The device provides separate body mass readings for different segments of the body and uses an algorithm incorporating impedance, age and height to estimate the total and regional body fat and fat-free mass. Total fat, lean mass percentages and the ALM were calculated using standard formulas (21). Sarcopenic obesity was defined based on the definition of Oh and colleagues ((ALM/weight) × 100%), which is less than 23.40 and 29.60 in females and males, respectively (18).

Daily steps were measured by means of a validated commercial grade pedometer (Omron HJ-320; Omron Healthcare Co., Ltd., Kyoto, Japan), which is considered accurate within ±5% of the criterion measure and was used to record the total number of steps taken each day, with the device automatically resetting itself at the end of the day and possessing a 7-day memory (22). The pedometer was placed either in participants' trouser pocket or attached to their waistband. Participants were encouraged to wear the pedometer all day for 7 days once awake and to remove the device only when sleeping or bathing and they were advised to continue with their usual lifestyle. A step-defined sedentary lifestyle index is <5,000 steps/day (23).

Cardiometabolic disease in this study is defined as the presence of any diseases such as type 2 diabetes, cardiovascular diseases (coronary heart disease, stroke, transient ischaemic attack, and peripheral arterial disease) and dyslipidaemia (a decreased concentration of high-density lipoprotein cholesterol and an increased concentration of high-density lipoprotein cholesterol and triglycerides) based on self-reported diagnosis, either simultaneously or separately.

### Statistical Analysis

Descriptive statistics were calculated as means and standard deviations, frequencies and proportions. A χ^2^ test and student's *t*-test were used to compare proportions and means, respectively, between participants with and without SO in the clinical sample. Simple and linear regression analysis was performed to calculate changes in the total daily steps among individuals with SO. A multiple linear regression model was adjusted for age, sex, employment, and the presence of cardiometabolic disease. All analyses were performed using SPSS version 25.0 (IBM Corp.; IBM, Armonk, NY, USA). Statistical significance was considered to be *p* < 0.05.

## Results

Table 1 describes socio-demographic characteristics of the study population, which included 111 participants with obesity with a mean age of 37.12 ± 15.58 years [32 males (28.8%) and 79 females (71.2%)]. Application of the chosen definition of SO (18) indicated that a total of 55 patients (49.5%) had SO, of whom 17 were male (53.1%) and 38 were female (48.1%). On the other hand, 56 (50.5%) did not have SO, of whom 15 were male (46.9%) and 41 were female (51.9%) (Table 1).

**Table 1:** Socio-demographic and anthropometric body composition characteristics of the study population (*n* = 111).

	**Total (*n* = 111)**	**Non SO (*n* = 56)**	**SO (*n* = 55)**	**Significance**
Age	37.12 (15.58)	34.67 (14.30)	39.62 (16.55)	0.095
Sex				X^2^ = 0.230, *p* = 0.632
Male	32 (28.8)	15 (26.8)	17 (30.9)	
Female	79 (71.2)	41 (73.2)	38 (69.1)	
Marital status				X^2^ = 8.728, *p* = 0.003
Unmarried	52 (46.8)	34 (60.7)	18 (32.7)	
Married	59 (53.2)	22 (39.3)	37 (67.3)	
Level of education				X^2^ = 0.216, *p* = 0.642
Lower education	67 (60.4)	35 (62.5)	32 (58.2)	
University	44 (39.6)	21 (37.5)	23 (41.8)	
Employment				X^2^ = 0.489, *p* = 0.484
Unemployed	67 (60.4)	32 (57.1)	35 (63.6)	
Employed	44 (39.6)	24 (42.9)	20 (36.4)	
BMI (kg/m^2^)	36.27 (5.13)	34.53 (4.31)	38.05 (5.33)	<0.001
FM (kg)	39.48 (11.50)	33.79 (7.37)	45.28 (12.10)	<0.001
FM (%)	40.22 (6.09)	36.88 (4.52)	43.62 (5.60)	<0.001
FFM (kg)	57.79 (12.23)	58.01 (11.24)	57.56 (13.27)	0.845
FFM (%)	59.65 (6.31)	63.13 (4.51)	56.11 (5.92)	<0.001
ALM by weight^*^100 (%)	24.96 (3.53)	26.61 (3.38)	23.28 (2.85)	<0.001
Daily steps	6,012 (2,903)	6,732 (2,989)	5,279 (2,641)	0.008
Cardiometabolic disease				X^2^ = 4.081, *p* = 0.043
Yes	20 (18.0)	6 (10.7)	14 (25.5)	
No	91 (82.0)	50 (89.3)	41 (74.5)	
		Males	Females	X^2^ = 0.230, *p* = 0.632
Non SO (n,%)	56 (50.5)	15 (46.9)	41 (51.9)	
SO^§^ (n,%)	55 (49.5)	17 (53.1)	38 (48.1)	

*BMI, Body Mass Index; FM, Fat mass; FFM, Fat free mass; ALM, Appendicular Lean Mass*;

§ *SO, sarcopenic obesity as defined by Oh et al. (18)*.

**Figure 1:**
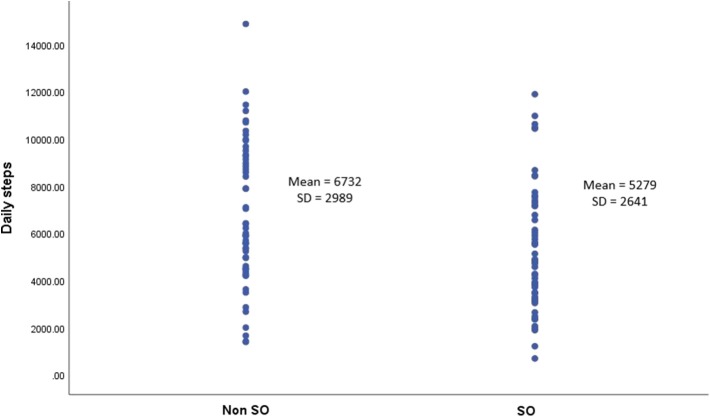
Mean daily steps among patients with or without SO (*n* = 111).

**Figure 2:**
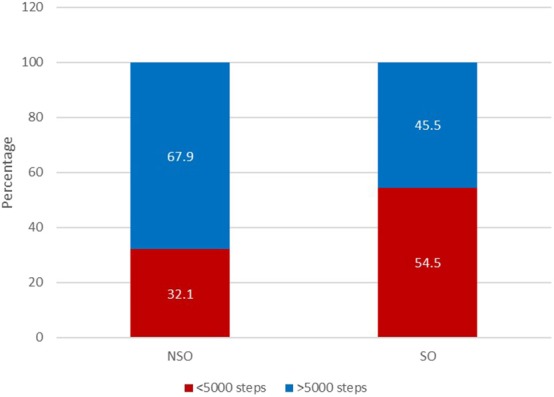
Proportion of patients with SO considered inactive, performing less than 5,000 steps daily (*n* = 111).

The group with SO, when compared with the group without SO, had a significantly higher BMI, total body-fat percentage and lower fat-free mass percentage as well as ALM/weight × 100 and a higher prevalence of cardiometabolic disease (25.5% vs. 10.7%) (Table 1). Moreover, the SO group had a lower mean number of daily steps than those in the group without SO (5,279 ± 2,641 vs. 6,732 ± 2,989; *p* = 0.008) (Table 1; Figure 1) and a significantly higher prevalence of inactive individuals (i.e., <5,000 daily steps) than those in the group without SO (54.5% vs. 32.1%; *p* = 0.017) (Figure 2). Linear regression analysis showed that having SO is associated with a lower number of daily steps by 1,421 (β = −1421.4; −2508.9, −333.9; *p* = 0.011) after adjusting for age, gender, employment and the presence of cardiometabolic diseases.

## Discussion

Our study aimed to provide data on the prevalence of sarcopenia in treatment-seeking adults with obesity and to assess any association between SO and a more sedentary lifestyle, expressed as measured routine daily steps. In turn, two major findings were revealed.

Firstly, our assessment of the prevalence of sarcopenia among patients with obesity seeking treatment, using criteria accounting for body weight, revealed an SO prevalence of 49.5% across the entire sample (53.1% in males and 48.1% in females). This falls within the large prevalence range of 0–100% reported for males and 0–85% reported for females, a range that may depend on the SO definition applied (24), in which a greater prevalence may be likely to be reported in studies accounting for body weight or BMI, whereas a smaller prevalence will be reported in those that do not (16). However, specifically, the prevalence of sarcopenia in our sample (53.1% in males and 48.1% in females) highly exceeded that found by Oh and colleagues in their original sample (19.6% in males and 31.3% in females) (18). Several factors may underlie this discrepancy, the sample in Oh and colleagues studies was composed of normal and overweight as well as obese individuals from the general population (18), unlike the present study, which included patients exclusively with obesity (BMI ≥ 30 kg/m^2^) in a clinical setting and affected by obesity-related (i.e., cardiometabolic) diseases. We speculate that obesity as well as cardiometabolic disease may act synergistically to increase the inflammatory status (25), when the latter is known to have a role in sarcopenia pathogenesis (8). In other words, concomitant cardiometabolic complications could increase the prevalence of sarcopenia in obesity and this can partially explain such a high prevalence of sarcopenia in our young population. However, we cannot exclude the existence of other factors (i.e., ethnicity) that may explain the higher prevalence of SO in our sample.

Second, 54.5% of participants with SO were considered inactive (i.e., <5,000 daily steps), with both conditions strongly associated; in fact, the presence of SO decreased the number of daily steps by 1,421 after adjusting for age, gender and employment. However, the cross-sectional design, at best, reveals only simple associations between SO and reduced mean daily steps but cannot provide firm information on any causal relationships between the two conditions (1, 26), in other words, whether SO causes a sedentary lifestyle or whether a tendency to be physically inactive over time causes SO.

Some clinical implications can be derived from our findings given the need to raise awareness among clinicians (and patients) of the presence of sarcopenia in those seeking treatment for obesity. Second, our results reveal the importance of screening for SO in treatment-seeking patients with obesity, since this condition seems to be associated with a more sedentary lifestyle and may have an impact on the effectiveness of weight management programmes based on lifestyle modification work, focusing on enabling patients to cope with sedentary behavior and helping them to develop a more active lifestyle (27–37).

Our study has certain strengths. Firstly, to the best of our knowledge, it is the first study to examine the association between SO and a sedentary lifestyle (i.e., daily steps), based on physical activity measurements using an objective, validated and accurate tool suitable for assessing lifestyle (22) rather than self-reported data, which has never been previously investigated. Second, it is one of the few to assess SO in the MENA region and one of the few studies that takes into account not only ALM but also body mass (18). Second, our results are based on physical activity measurements using an objective, validated and accurate tool suitable for assessing lifestyle (22) rather than self-reported data.

However, the study has certain limitations. Firstly, our results need to be interpreted with caution because they may not applicable to patients treated in other settings (i.e., inpatients or bariatric surgery). Second, we assessed body composition using an impedance analyser; despite being validated, this has not yet been accepted as a gold-standard technique for patients with obesity (38). Third, the cross-sectional design, small sample size and the absence of macronutrient nutritional and biochemical assessments in our study, which permit a better understanding of the mechanisms underlying the high prevalence of sarcopenia in individuals with obesity, in addition, the use of a definition for SO that was initially established in an Asian population, that was based only on reduction in LBM, not taking into account low muscle strength or low physical function, should be considered further limitations. Finally, in the diagnosis of cardiometabolic disease, we relied on self-reported data.

## Conclusions

Our findings provide preliminary evidence that nearly 50% of adults with obesity, who are seeking weight-loss treatment, have sarcopenia. This condition seems to be strongly associated with a more sedentary lifestyle. It would, therefore, be clinically useful to screen SO in this population. However, future longitudinal studies are needed to clarify whether this may influence clinical outcomes, namely weight loss and maintenance as well as dropout rates. If this is confirmed, weight management programmes should take into consideration additional physical activity strategies in this population to promote the adoption of more active lifestyles.

## Data Availability Statement

The datasets generated for this study are available on request to the corresponding author.

## Ethics Statement

The studies involving human participants were reviewed and approved by Institutional Review Board—Beirut Arab University. The patients/participants provided their written informed consent to participate in this study.

## Author Contributions

All authors claim authorship, and have approved and made substantial contributions to the conception, drafting and final version of the paper. This study was designed by ME, while LI conducted the statistical analysis. DK, DE, and HT collected data. ME and LI co-wrote the manuscript.

### Conflict of Interest

The authors declare that the research was conducted in the absence of any commercial or financial relationships that could be construed as a potential conflict of interest.
